# Salivation Treated by Nitrate of Silver

**Published:** 1850-03

**Authors:** 


*Salivation Treated by Nitrate of Silver.* By —— Kirby, Esq.—In a late number of the Medical Press, M. Bouchacourt is reported to have used successfully a solution of nitrate of silver in a case of profuse salivation, in which various gargles and sinapisms, &c., have been employed without benefit. I have had much experience of this remedy, having used it for many years in cases of sudden and inordinate salivation. It rapidly arrests stomatitis and the attendant profuse discharge, provided it be applied of proper strength and with sufficient frequency. Of course the teeth become deeply discolored, but these stains being removable by means which shall be noticed, temporary disfigurement is not to be urged as an objection to a free and general employment of so controlling a remedy.

*A Case Selected as an Example.*—Mr. G., under treatment for bronchitis, with engorgement of the liver and heart, was unexpectedly seized with violent salivation and distracting pain of his gums, which were unusually swollen. I ordered the following solution:

℞. Nitratis argenti drachmam. Aque distillate unciam. Sol.

This solution was freely applied three or four times daily, with a hair pencil, by my friend Mr. Collins. The heat and smarting which ensued upon each application quickly subsided, and was followed by a period of much ease. In three days the patient's mouth was well.

Until now he had no idea of the condition of his teeth, of whose shape and color he was always rather vain. His annoyance and dissatisfaction at the "ruinous" change of these adornments was displayed in terms of no equivocal import; his chagrin, however, was soon allayed as he saw them brighten under the use of a solution of hydriodate of potash, in the proportion of two drams to an ounce of distilled water; which solution was frequently applied by a brush. He now left for the country, and continued this treatment. When I saw him on his next return to town, his teeth had nearly resumed their former attractive lustre; I say nearly, for although darkness had wholly disappeared, yet a slightly yellowish tinge was perhaps observable.

Mr. Collins, who has been engaged in some experiments as to the means by which stains from the nitrate of silver may be most effectually removed, has favored me with the following report: "I applied a solution of nitrate of silver, in the proportion of a drachm to an ounce of distilled water, to two extracted teeth for some days, until a deep blackness was produced. I treated one by a solution of the cyanide of potash, in the proportion of one part of the salt to two of distilled water; the other I treated by a solution of hydriodate of potash, in the proportion of four grains to ten minims of distilled water; and the hyposulphite of potash in the proportion of one part of the salt to two of distilled water. The latter plan succeeded very well, but the former produced a much better effect, more thoroughly removing all traces of discoloration, after being patiently and carefully applied by means of a soft brush.—*Dublin Med. Press, August* 1, 1849, *p.* 70.

[Mr. Collins, in another number of the Dublin Medical Press gives the following further information on the subject:]

If a solution of cyanide of potassium be added to a solution of nitrate of silver, a whitish precipitate of cyanide of silver immediately occurs. This precipitate is perfectly soluble in an excess of the cyanide of potassium, forming a colorless solution. Black stains produced by nitrate of silver on the skin, nails, teeth, and on linen, &c., brushed over with a solution of the cyanide of potassium (eight or ten grains of the salt to ℈j of distilled water) are similarly acted on. They will disappear after one or two applications if at all superficial; if even deeply seated in the textures they may require several. The cyanide of potassium is a cheap salt, and may be had at the Apothecaries' Hall, or from any respectable druggist. The solution should be made fresh, as it is liable to spontaneous decomposition.—*Dublin Medical Press, August* 8, 1849, *p.* 83.

